# Factors Hindering Social Participation among Older Residents from Evacuation Zones after the Nuclear Power Plant Accident in Fukushima: The Fukushima Health Management Survey

**DOI:** 10.3390/ijerph18094426

**Published:** 2021-04-21

**Authors:** Mayumi Harigane, Hiromi Imuta, Seiji Yasumura, Fumikazu Hayashi, Hironori Nakano, Tetsuya Ohira, Masaharu Maeda, Hirooki Yabe, Yuriko Suzuki, Kenji Kamiya, on behalf of the Mental Health Group of the Fukushima Health Management Survey

**Affiliations:** 1 Radiation Medical Science Center for the Fukushima Health Management Survey, Fukushima Medical University School of Medicine, Fukushima 960-1295, Japan; yasumura@fmu.ac.jp (S.Y.); fhayashi@fmu.ac.jp (F.H.); h-nakano@fmu.ac.jp (H.N.); teoohira@fmu.ac.jp (T.O.); masagen@fmu.ac.jp (M.M.); kkamiya@fmu.ac.jp (K.K.); 2 Department of Public Health, Fukushima Medical University School of Medicine, Fukushima 960-1295, Japan; yrsuzuki@gmail.com; 3 Faculty of Health Sciences, Tokyo Metropolitan University, Tokyo 116-8551, Japan; h_imuta@tmu.ac.jp; 4 Department of Epidemiology, Fukushima Medical University School of Medicine, Fukushima 960-1295, Japan; 5 Department of Disaster Psychiatry, Fukushima Medical University School of Medicine, Fukushima 960-1295, Japan; 6 Department of Neuropsychiatry, Fukushima Medical University School of Medicine, Fukushima 960-1295, Japan; hyabe@fmu.ac.jp; 7 National Center of Neurology and Psychiatry, Department of Mental Health Policy, National Institute of Mental Health, Tokyo 187-8553, Japan; 8 Research Institute for Radiation Biology and Medicine, Hiroshima University, Hiroshima 734-8553, Japan

**Keywords:** social participation, older adults, evacuation zone, Japan, Fukushima prefecture, activities of daily living, Great East Japan Earthquake, Fukushima Health Management Survey

## Abstract

Considering the health effects of radiation accompanying the nuclear power plant accident that occurred in the wake of the Great East Japan Earthquake, this study aimed to examine social participation after the disaster and factors hindering participation among citizens aged ≥ 65 years from designated evacuation zones inside the Fukushima prefecture. The target population comprised 180,604 residents in 13 municipalities containing designated evacuation zones. There were 73,433 valid responses (response rate, 40.7%); of which, data from 19,573 respondents aged ≥ 65 years were analyzed. Multinomial logistic regression analyses were conducted to investigate the factors associated with social participation. In total, 53.0% of older evacuees did not participate in recreational activities or communal services. Stratified analysis showed that living outside the Fukushima prefecture and requiring assistance with activities of daily living were associated with low social participation. This study clarified that the majority of older evacuees did not participate in social activities at the time of the survey within one year of the disaster. Furthermore, where these older individuals were evacuated to and whether they were able to live independently might have affected their social participation. Better subjective health, better sleep quality, and more frequent exercise may be associated with improved social participation.

## 1. Introduction

The Great East Japan Earthquake and the resulting tsunami of 11 March 2011 led to a nuclear incident at TEPCO’s Fukushima Daiichi Nuclear Power Station. Psychosomatic abnormalities have been indicated as a key long-term health effect of the Chernobyl Nuclear Accident [1,2]. Many residents of the Fukushima prefecture have experienced psychological distress caused partly by anxiety over radiation and evacuation, the deaths of relatives, the loss of homes and other property, and other frightening experiences [3,4]. Mobility problems, difficulties with self-care, difficulties with daily living, and mood disorders have been found to have a negative impact on quality of life (QOL). It has been confirmed that economic or material assistance, as well as social support, is necessary for improving QOL [5].

Residents who were forced to evacuate their homes have been separated from the communities to which they were accustomed and are now living in new and unfamiliar areas. Since people who evacuated to areas outside the Fukushima prefecture were shown to have higher levels of psychological distress, in comparison to those from other communities within the same prefecture [6], it is predictable that they will take more time to adapt to their new environment. Since it was shown that social networks become smaller with age after adolescence [7,8,9], it can be predicted that evacuees aged ≥ 65 years would be likely to have difficulty in starting new social participation in a strange area after evacuation, even if they were socially active before the disaster. Decreased participation in social activities has been noted as a risk factor for the onset of “disuse syndrome,” which frequently affects older people in the wake of disasters [10], and promoting participation in social activities is considered to be an effective preventive measure. The benefits of social participation among older adults reportedly include positive impacts on subjective well-being and health perceptions, as well as enhanced motivation and sense of purpose [11,12]. Hence, clarifying the frequency of social participation among older residents of evacuation zones who were relocated to areas both inside and outside the Fukushima prefecture, as well as exploring the factors associated with such participation, is important from both a care-prevention perspective and for identifying specific measures. However, very few studies, both in Japan and overseas, have evaluated social activities among older evacuees in the first year after disasters.

The Mental Health and Lifestyle Survey, part of the Fukushima Health Management Survey, was administered in the Fukushima prefecture to obtain an accurate understanding of the physical and mental health (and other health problems) of all residents in the evacuation area after the disaster. In addition to providing proper care related to their welfare, the survey also aimed to benefit future generations with better mental care and support during emergencies or national disasters [3,13].

Thus, based on the data from the Fukushima Health Management Survey, the aim of this study was to investigate the following hypotheses regarding the frequency of social participation among older adults who had been relocated from evacuation zones:

1. The low frequency of social participation among older adults may be related to relocation to areas outside the Fukushima prefecture and requiring assistance for activities of daily living (ADL).
2. Factors hindering social participation may vary according to the evacuation location.
3. Factors hindering social participation may vary according to whether the older evacuees required assistance for ADL.

## 2. Materials and Methods

### 2.1. Survey Participants

A total of 180,604 residents of the 13 municipalities containing evacuation zones designated by the national government on 11 March 2011, were asked to complete the 2011 FY “Mental Health and Lifestyle Survey” (for general use), which was a part of the Fukushima Health Management Survey. The areas targeted by the survey included evacuation zones (districts associated with the former designated locations recommended for evacuation) in the cities of Minamisoma and Tamura; the towns of Kawamata, Hirono, Naraha, Tomioka, Okuma, Futaba, and Namie; and the villages of Kawauchi, Katsurao, and Iitate; as well as part of the city of Date. The overall number of valid responses received was 73,433 (response rate, 40.7%). For this study, survey data of 19,573 respondents who responded by themselves and were aged ≥ 65 years (9048 men and 10,525 women) were selected for analysis. The average age of these respondents was 74.6 ± 6.0 years, with an age range of 65–101 years.

The survey was administered from 30 January 2012, to 31 October 2012, and the questionnaires were sent to the recipients via post. The questions taken from the Mental Health and Lifestyle Survey (for general use) included information on the age, sex, and highest education level of the respondents and whether they answered in person or by proxy. Questions were asked regarding social participation in recreational activities (e.g., karaoke and gateball) and community activities (e.g., local festivals). The three possible responses were “Never or rarely participate,” “Sometimes,” or “Often.” Regarding disaster-related items, questions about current domicile (evacuation shelter, temporary housing, rental housing/apartment, relative’s home, own home, and other), present evacuation location (inside the Fukushima prefecture/outside the Fukushima prefecture), experience of the Great East Japan Earthquake (earthquake, tsunami, nuclear power plant accident (e.g., hearing the explosion)), results of the government’s building damage assessment, presence or absence of changes in work due to the earthquake, and whether or not the respondents were personally bereaved by the earthquake were also asked. To evaluate physical and mental health, items related to subjective health, presence or absence of mental illness, ADL, sleep disorders (seven items), and frequency of exercise were included. For ADL, respondents were asked whether they required assistance with eating, clothing, using the toilet, and shopping. The analyses were conducted by allocating those who required assistance with any of these four activities into the “ADL assistance required group” and those who did not require assistance for any of these activities were allocated into the “ADL-independent group.” Furthermore, the Kessler Psychological Distress Scale (K6) [14,15] and Post-Traumatic Stress Disorder Checklist (PCL-S) [16] were used to evaluate mental health. The K6 consists of six items to assess psychological distress. Scores range from 0 to 24 points, with higher values indicating higher levels of psychological distress. Based on a previous study [17], those who scored between 13 and 24 were categorized as “≥13” and those who scored between 0 and 12 were categorized as “<13.” The PCL-S consists of 17 items that evaluate traumatic reactions. Scores range from 17 to 85 points, with higher values indicating higher levels of traumatic reactions. Based on previous studies [16,18], those who scored between 44 and 85 were categorized as “≥44” and those who scored between 17 and 43 were categorized as “<44.”

### 2.2. Data Analysis

To examine the relationship with the frequency of social participation, chi-square tests were performed for categorical data such as age and sex, and *t*-tests were performed for continuous variables. Furthermore, logistic regression analyses were conducted, in which age and sex were adjusted using the explanatory variables that showed statistically significant associations with social participation in the univariate analyses. Analyses were performed using SAS 9.4 (SAS Institute Inc., Cary, NC, USA) statistical software package, with the significance level set at 5% (on either side).

### 2.3. Ethical Considerations

We explained in writing to the participants that responses would not be published in any form that identified individuals. Participants who answered the self-report questionnaires were considered to have consented to participate. This study was approved by the Ethics Committee of Fukushima Medical University (1316).

## 3. Results

### 3.1. Frequency of Social Participation

Regarding the frequency of participation in social activities, 10,375 (53.0%) respondents answered that they did not participate or rarely participated, 6538 (33.4%) answered that they occasionally participated, and 2660 (13.6%) answered that they often participated in such activities. Respondents were allocated into two groups: social nonparticipation group (did not participate or rarely participated) and social participation group (occasionally or often participated).

### 3.2. Associations between the Participants’ Characteristics, Disaster-Related Items, and Physical and Mental Health by Frequency of Social Participation

Table 1 shows the participants’ basic characteristics and disaster-related items of each social participation group. Significant differences were observed in age and sex between the groups. Female participants and older participants were less likely to engage socially. Regarding the disaster-related items, significant differences were found regarding evacuation location, experience of the tsunami, the result of domicile earthquake damage assessment, and earthquake-related bereavement. The strongest association was found among respondents who relocated to areas outside the prefecture. This was associated with lower social participation as well as not having experienced the tsunami, having received an earthquake damage assessment of large-scale partial or complete destruction, and having experienced earthquake-related bereavement.

The relationships between the participants’ reported physical and mental conditions and frequency of social participation were significant for all the items (Table 1). Those in the social nonparticipation group tended to assess their subjective health as poor or extremely poor. This group was also characterized by more frequent reports of mental illness as well as a large number of respondents who required assistance with ADL. With respect to sleep satisfaction, those who reported that they were dissatisfied tended to be found in the social nonparticipation group. Among the items related to sleep disorders, those who participated less often in social activities were more likely to report taking a long time to fall asleep at night, awakening suddenly during the night, waking up earlier than desired, being unable to go back to sleep, sleep deprivation, daytime feelings of depression, decline in mental and physical activity during the day, and daytime drowsiness. Respondents who answered that they exercised rarely were more likely to be in the social nonparticipation group.

The results of chi-square tests for PCL and K6 scale by frequency of social participation showed that both scores were significantly higher in the social nonparticipation group (Table 1). A higher likelihood of experiencing trauma or psychological distress was found among older respondents who did not engage in social activities.

### 3.3. Factors Associated with Social Participation

Table 2 shows the results of the binary logistic regression analysis with social nonparticipation as the dependent variable. After introducing social participation, a multiple logistic regression analysis was performed on the items that showed univariate significance using a stepwise method, adjusting for age and sex.

Among the disaster-related items, evacuation to an area outside Fukushima was linked to a 2.09 times higher likelihood, experience of the tsunami was linked to a lower likelihood (AOR 0.77), and having their house fully destroyed (as compared with experiencing no damage or only partial damage) was linked to a 1.69 times higher likelihood of social nonparticipation. Among the items related to physical and mental health, requiring assistance for ADL was associated with a 3.85 times higher likelihood, evaluating one’s own health as extremely poor (compared with those who selected other options) was associated with a 2.02 times higher likelihood, every one-point increase in the AIS score was associated with a 1.08 times higher likelihood, and rarely or never exercising (compared with those who exercised almost every day) was associated with a 2.74 times higher likelihood of social nonparticipation. The results were in line with our hypothesis that evacuation location and the requirement of assistance for ADL were factors hindering social participation.

### 3.4. Factors Associated with Social Participation by Evacuation Location

We investigated the differences between factors hindering social participation among older evacuees who relocated outside the Fukushima prefecture compared with those who relocated within the Fukushima prefecture.

Factors associated with social participation among evacuees who relocated within the Fukushima prefecture are shown in Table 3. Among disaster-related items, it was found that those who experienced the tsunami were 26% less likely to have hindered social participation, while those whose houses were fully destroyed (as opposed to those who experienced no damage or only partial damage) were 76% more likely to have hindered social participation. Regarding items related to physical and mental health, those who required assistance with ADL (as compared with those who were independent) were 4.50 times more likely and those who evaluated their health as poor (compared with those who evaluated their health as good) were 2.02 more likely to have hindered social participation. Furthermore, it was found that every one-point increase in the AIS score, having 13 or more points on the K6 scale, and never or rarely exercising (compared with those who reported exercising almost every day) increased the likelihood of hindered social participation by 1.08 times, 1.24 times, and 2.79 times, respectively.

Table 4 shows the factors related to social participation among those who relocated to areas outside the prefecture. None of the disaster-related items were significant deterrents for social participation among the out-of-prefecture residents. Regarding the health-related items, it was found that requiring some form of assistance with ADL increased the likelihood of hindered social participation by 1.97 times, and never or rarely exercising (versus exercising almost every day) increased the likelihood by 2.73 times.

### 3.5. Factors Associated with Social Participation by Activities of Daily Living

Table 5 and Table 6 show the results of the differences in the factors hindering social participation between the ADL-independent and ADL-assistance required groups.

In the ADL-independent group, three disaster-related items were shown to be risk factors for hindered social participation. Among the disaster-related items, relocation to an area outside the Fukushima prefecture increased the likelihood of social nonparticipation by 2.13 times, and complete or large-scale partial destruction of a house increased the likelihood by 1.79 times; however, first-hand experience of the tsunami reduced the likelihood by 22%. It was found that the likelihood of social nonparticipation increased by 2.01 times among those who evaluated their own health condition as extremely poor, by 1.08 for every one-point increase in the AIS score, and by 2.74 times among those who never or rarely exercised (versus those who reported exercising almost every day) (Table 5).

In the ADL assistance required group, none of the disaster-related items were found to be significantly related to factors that hindered social participation. Among the items related to physical and mental health, it was found that the likelihood of social nonparticipation increased by 3.31 times among those who evaluated their own health condition as extremely poor and by 3.14 times among those who rarely or never exercised (versus those who reported exercising almost every day) (Table 6).

## 4. Discussion

This study focused on data of older residents aged ≥ 65 years in designated evacuation zones after the Great East Japan Earthquake to examine the current state of social participation after the earthquake and factors hindering such participation in the first year following the disaster. Although many previous studies have investigated mental and physical health, including perspectives such as QOL and trauma [2,3,4,5], there has been a lack of research on social participation in the first year following a disaster.

Our hypotheses regarding the associations of evacuation location and ADL with social participation were proven, i.e., evacuation to a place outside the Fukushima prefecture and requiring assistance for ADLs (such as eating, dressing, using the toilet, and shopping) were found to have hindered social participation among older participants after the disaster. Furthermore, our findings showed that factors hindering social participation varied by both evacuation location and whether assistance was required for ADL.

According to previous studies [7,8,9], social networks tend to become smaller after adolescence. For older people, familiar relationships, such as family and neighborhood friends, become the center of social networks. In our previous study [6], it was found that evacuation after the nuclear accident to a site outside of the Fukushima prefecture resulted in more psychological distress compared to sites inside of the Fukushima prefecture. However, this difference was reduced by providing support for social isolation, loss of work, and problem-drinking behavior [6]. A study on the neighborhood relationships and community participation of the elderly living in housing complexes in suburban communities [19] found that the number of neighborhood friends and the number of trustworthy friends were the important determinants of social participation. These findings suggest that when the elderly are separated from their families and neighborhood friends due to disasters, their social networks become dramatically smaller. As a result, even those who have been able to participate in society by interacting with those around them are likely to decrease their social participation. In addition, there is a strong association between low independence in ADLs and low frequency of going out [20], which is consistent with the fact that older people who needed help with ADLs in this study had less social participation than those who were independent. Therefore, it would be useful for health care professionals, such as public health nurses, to actively promote social participation by the elderly people who have evacuated to other prefectures. Moreover, it is necessary to actively encourage the elderly who need assistance with ADLs to participate in social activities; it might be possible to maintain and improve their ADLs, and enhance their well-being through social participation, by encouraging them to participate in programs that prevent the decline of ADLs.

### 4.1. Situation of Social Participation among the Study Participants

There is no consistent definition of social participation in old age used by studies conducted in Japan and overseas [10,11,21]. In this study, social participation was regarded as participation in recreational activities (e.g., karaoke, gateball) and community services (e.g., local festivals). Using this definition, the social participation frequency among older participants from evacuation areas in the Fukushima prefecture was 47%. Although not directly comparable as they were not assessed in the same way, the frequency of social participation was over 70% in a previous study of community-dwelling elderly people in Japan, which defined social participation as being involved in one or more of the following activities: hobbies and other activities among peers, neighborhood association/resident association activities, senior citizens’ club activities, and volunteer activities [22]. More than 60% of the respondents participated in one or more community organizations in another previous study in Japan, the Aichi Gerontological Evaluation Study project, which investigated participation in one or more of the following eight types of organizations: political organizations, industry associations, volunteer activities, civic movements, religious organizations, sports clubs, neighborhood and senior citizens’ clubs, and hobby associations [23]. More than 70% also participated in collective activities in the Berlin Aging Study, which asked interviewees if they had participated in 11 activity domains (hobby, traveling, day trips, sports, culture, games, education, art, dancing, voluntary social engagement, and politics) during the year before the interview; the interviewees were prompted to describe their specific engagements and their activities were classified as “voluntary social engagement” and “politics” first, and the remaining activities were classified as “collective participation” or “no social participation” using information from their contexts [24]. The participation in collective activities was 77% in the baseline data and 88% in the follow-up data [24].

In these previous studies, the definition of social participation was more detailed and extensive than in the present study. Thus, there might have been more elderly people that answered affirmatively when asked about social participation in the present study; however, it is difficult to say that it was equal to or greater than that reported in previous studies. From these results, it can be assumed that following the earthquake, many people were occupied with rebuilding their lives and were therefore no longer able to participate in social activities. Incidentally, a study that surveyed outdoor activities among older residents forced to evacuate after a typhoon in Taiwan in 2009 found that community cohesion had a positive effect on outdoor activities [25]. Considering possible cultural differences between Taiwan and Japan, and that nonrespondents to this survey accounted for approximately 60% of the older evacuees, we must be cautious in our interpretations. However, our results showed that about half of the participants were engaging socially; therefore, adopting measures to collaborate with and support residential communities at the evacuation locations should be done to continue that level of participation.

### 4.2. Factors Associated with Social Participation

Subjective health and frequency of exercise were common factors across all the analyses that were significantly associated with social participation. The association between subjective health and social activities has been verified previously in other studies [11,12]. Self-rated health, which is synonymous with subjective health, is affected by physical health, mental health, and social factors [26]. Self-rated health is also a comprehensive health indicator that reflects both objective physical health and mental health and has been shown to have associations with diseases, sense of well-being, participation in social activities, and interpersonal contact [27]. The association of social participation with subjective health was confirmed again in this study.

Our results found that participants who exercised less were less likely to participate in social activities. In the 2013 National Health and Nutrition Survey [28], individuals who engaged in habitual exercise accounted for 50% of men and 40% of women over the age of 70. In this context, “individuals who engage in habitual exercise” referred to people who exercised for 30 min or more at least twice a week over the course of at least one year. Although direct comparison is not possible, given that the results of this study did not measure the duration of a single session of exercise or duration of exercise habits, individuals who exercised at least twice a week accounted for 68.6% of the social participation group and 49.8% of the social nonparticipation group. These results showed that many participants exercised after the disaster. As exercise is an activity that can be accomplished either at home or outdoors, it can be inferred that many older evacuees made a conscious decision to incorporate exercise into their daily routines following the disaster. Walking paths, parks, fitness facilities, and other equipment and infrastructure were damaged during the Great East Japan Earthquake [29]. Owing in part to the fact that this study was particularly concerned with the first year following the disaster, it may be inferred that many people who had previously engaged in social participation chiefly through physical activities outdoors were unable to do so as they found it difficult to find a suitable location in their present evacuation locations.

In a study that examined changes in the physical activity levels over a one-year period among evacuees of the Great East Japan Earthquake living in temporary housing in the Iwate prefecture, it was reported that medium-intensity physical activity levels increased among older residents who were still comparatively younger [29]. Conversely, the occurrence of heart failure after an earthquake was more pronounced among individuals aged ≥ 75 years [30]. It was clear that the physical and mental impacts of disasters had serious consequences, especially among older individuals. Long-term periods of inactivity are associated with an increased risk of mortality and the onset of a variety of diseases. Hence, focused support is needed for older people who are physically inactive and are likely to have difficulties with increasing their physical activity levels to ensure that long-term inactivity does not occur [29]. It may be unlikely that older evacuees who exercise very little and have little physical strength will be able to engage in social activities. For this reason, from a long-term perspective, it is necessary to provide support that encourages people lead active lifestyles, such as by promoting exercise.

Between 20% and 40% of general adults reported having some kind of sleep disorder, and insomnia among Japan’s older population has become a health issue that can no longer be ignored [31]. In this study, it was observed that the lower the quality of sleep, the lower the likelihood of participating in social activities. In addition, from a longitudinal study of social participation and adult sleep patterns, Chen et al. revealed that individuals who were actively involved in social activities were able to sleep well [32]. However, they were not able to demonstrate that increased participation in social activities resulted in improvements in sleep. As this study was not a longitudinal one, causal relationships could not be demonstrated.

### 4.3. Disaster Experience, Evacuation Location and ADL, and Social Participation

Differences were observed in items associated with social participation and differences due to evacuation location. The evacuees who relocated to areas inside the prefecture were characterized by age, experience of the tsunami, extent of house damage, subjective health, psychological distress, and quality of sleep. However, no significant association was found among evacuees who relocated to areas outside the prefecture. The need for assistance with ADL and the frequency of exercise showed significant associations with social participation for both types of evacuees whether they relocated to areas inside or outside the prefecture. The factors related to social nonparticipation were found to be the need for assistance with daily functions and a low level of exercise among evacuees in the prefecture. Although not shown in the results, the average score on the K6 scale was 6.00 among evacuees inside the prefecture, while the average score for those who had been relocated to areas outside the prefecture was 7.29. From a mental health survey conducted among adults aged ≥ 18 years in the town of Ōtsuchi in the Iwate prefecture after the Great East Japan Earthquake, the average score on the K6 scale for the 65- to 74-year-old age group was 6.04, while that for the ≥75-year-old age group was 7.00 [33]. These data suggest that psychological distress among evacuees who relocated to areas outside the prefecture was also profound. However, no association was found with social participation. In addition, compared with evacuees inside the prefecture, evacuees outside the prefecture included a high proportion of individuals with mental health impairments receiving telephone assistance [34], suggesting that the experience of evacuation had affected their lifestyles. This result needs to be interpreted carefully, pending the accumulation of longitudinal data.

### 4.4. Implications

To improve care for the elderly, it is important to clarify the frequency of social participation by older people evacuated after a disaster and to explore the associated factors. However, few studies have assessed the social activities of the elderly after disasters. The results of this study suggest that when drastic life changes occur, such as evacuation after a nuclear accident, the social participation by the elderly may decrease; this decrease in social participation is associated with relocation to distant places, such as a different prefecture, and a decrease in ADLs. Disasters such as earthquakes, floods, and hurricanes occur all over the world, and many people might be forced to evacuate during such disasters in the future. In such cases, the elderly will need more social resources to maintain their psychological and physical well-being. Therefore, health care professionals need to prioritize the promotion of social participation by the elderly, especially those who need ADL support and who have evacuated to a distant location far from where they lived before.

### 4.5. Limitations of the Study

The first limitation is the definition of social participation. Despite the large number of studies evaluating social participation, these investigations were conducted using a variety of definitions stemming from their respective research interests, resulting in the present lack of a single uniform definition, even within the given fields of study [21,24]. Furthermore, most studies have investigated the simple presence or absence of participation, and no research that considers the degree of participation or involvement has been carried out [21,24]. Social participation as treated in this study is a narrow concept, and we were not able to capture other forms of social participation. In the future, it will likely be necessary to carry out a follow-up study involving a broader definition of social participation to verify changes in social participation among older individuals taking place along with reconstruction.

Second, the possible responses for the social participation question used in this study were subjective; therefore, there is a possibility that the results would have been different had a more objective question on social participation been asked.

Third, the response rate in this survey was low (40.7%). Among those who did not return the questionnaire, many faced a variety of problems. In addition, as this was a cross-sectional survey, we were unable to determine the causality of our results. However, an ongoing survey, the “Fukushima Health Management Survey,” will soon reveal the factors that promote or inhibit social participation among older evacuees.

## 5. Conclusions

In the present study, it was found that in the first year after a disaster, social participation among evacuees aged ≥ 65 years was less than half. It was also found that factors hindering social participation varied by the evacuation location and degree of assistance required for ADL. These results suggest that it is necessary to actively encourage social participation among the elderly who are likely to be isolated due to drastic changes in their environment, such as the evacuees after a nuclear accident. Furthermore, poor subjective health, poor sleep quality, and lack of exercise hindered social participation. If health care professionals find elderly people with these characteristics after evacuation, they should assess whether they are participating in society and, in some cases, encourage them to participate more in society. In the future, conducting a follow-up survey is warranted to clarify the causal relationships between social participation and the associated items needed to implement specific measures that help promote social activities among older evacuees.

## Figures and Tables

**Table 1:** Participants’ basic characteristics, disaster-related items, and physical and mental health by frequency of social participation.

		Participation in Recreational and Community Activities				
		Often or Sometimes		Never or Rarely Participated		*p* Value
		*n*	%	*n*	%	
Age at the time of survey	65–74 years	5288	57.5	5324	51.3	<0.0001
	75–84 years	3499	38.0	4148	40.0	
	≥85 years	411	4.5	903	8.7	
Sex	Men	4504	49.0	4544	43.8	<0.0001
	Women	4694	51.0	5831	56.2	
Highest education level completed	Elementary school or junior high school	4003	46.3	4436	45.6	0.066
	High school	3615	41.8	4020	41.3	
	Vocational school or junior college	678	7.8	810	8.3	
	University or graduate school	354	4.1	467	4.8	
Evacuation location	Fukushima prefecture	8391	91.2	8526	82.2	<0.0001
	Other prefecture	807	8.8	1849	17.8	
Experience of the tsunami	Did not experience	6979	75.9	8077	77.9	0.001
	Experienced	2219	24.1	2298	22.2	
Experience of the nuclear power plant accident	Did not experience	3767	41.0	4109	39.6	0.055
	Experienced	5431	59.1	6266	60.4	
Result of the domicile earthquake damage assessment	No or some damage	7428	84.9	7904	81.7	<0.0001
	Partial destruction	713	8.2	830	8.6	
	Large-scale partial or complete destruction	609	7.0	943	9.7	
Earthquake-related bereavement	No	6868	78.5	7573	77.1	0.022
	Yes	1882	21.5	2250	22.9	
Changes in work	No changes	4319	54.0	4678	54.4	0.596
	Had changes	3687	46.1	3928	45.6	
Health condition	Extremely good, good	1386	15.6	849	8.5	<0.0001
	Normal	6008	67.5	6104	61.2	
	Extremely poor, poor	1503	16.9	3015	30.3	
History of mental illness	No	8347	96.2	9029	93.9	<0.0001
	Yes	331	3.8	590	6.1	
ADL (eating, getting dressed and undressed, using the toilet, going shopping, etc.)	Independent	8856	98.3	9060	90.2	<0.0001
	Assistance required	151	1.7	987	9.8	
Satisfaction with sleep quality	Satisfied	3183	46.6	2708	36.8	<0.0001
	Slightly dissatisfied	2744	40.2	3011	40.9	
	Quite dissatisfied	668	9.8	1180	16.0	
	Very dissatisfied or had not slept at all	229	3.4	458	6.2	
Sleep induction	<3 days per week	4604	55.2	4260	45.2	<0.0001
	≥3 days per week	3732	44.8	5166	54.8	
Awakening during the night	<3 days per week	2681	32.2	2328	24.6	<0.0001
	≥3 days per week	5638	67.8	7129	75.4	
Awakening in the early morning	<3 days per week	4332	53.9	4065	44.9	<0.0001
	≥3 days per week	3713	46.2	4983	55.1	
Total sleep duration insufficiency	<3 days per week	5496	71.3	5367	62.1	<0.0001
	≥3 days per week	2214	28.7	3270	37.9	
Well-being during the day	<3 days per week	6022	80.8	5635	67.4	<0.0001
	≥3 days per week	1427	19.2	2728	32.6	
Functioning during the day	<3 days per week	5519	71.5	5007	57.4	<0.0001
	≥3 days per week	2201	28.5	3723	42.7	
Sleepiness during the day	<3 days per week	4633	59.0	4335	48.5	<0.0001
	≥3 days per week	3225	41.0	4599	51.5	
Frequency of exercise	Almost every day	2649	30.3	2207	22.5	<0.0001
	2–4 times a week	3352	38.3	2673	27.3	
	About once a week	1474	16.9	1390	14.2	
	Rarely or never	1274	14.6	3520	36.0	
K6 score (Psychological distress)	<13	7076	89.4	7128	81.2	<0.0001
	≥13	842	10.6	1655	18.8	
PCL-S (Trauma reaction scale)	<44	6273	76.7	6300	69.7	<0.0001
	≥44	1901	23.3	2745	30.4	

**Table 2:** Results of the logistic regression analysis with social nonparticipation as the dependent variable.

Factor		Crude Odds Ratio	95% Confidence Interval		*p* Value	Adjusted Odds Ratio	95% Confidence Interval		*p* Value
Age	For every 1-year increase	1.03	1.03	1.04	<0.0001	1.02	1.01	1.03	<0.0001
Sex	Men								
	Women	1.23	1.16	1.30	<0.0001	1.12	1.02	1.23	0.021
Highest education level completed	Elementary school or junior high school								
	High school	1.00	0.94	1.07	0.91	0.98	0.89	1.09	0.697
	Vocational school or junior college	1.08	0.97	1.20	0.18	0.94	0.79	1.12	0.508
	University or graduate school	1.19	1.03	1.38	0.02	1.35	1.09	1.67	0.005
Evacuation location	Fukushima prefecture								
	Other prefecture	2.25	2.06	2.46	<0.0001	2.09	1.81	2.42	<0.0001
Tsunami	Did not experience								
	Experienced	0.90	0.84	0.96	0.00	0.77	0.68	0.87	<0.0001
Result of the domicile earthquake damage assessment	No or some damage								
	Partial destruction	1.09	0.99	1.22	0.09	1.07	0.90	1.28	0.448
	Large-scale partial or complete destruction	1.46	1.31	1.62	<0.0001	1.69	1.41	2.04	<0.0001
Earthquake-related bereavement	No								
	Yes	1.08	1.01	1.16	0.02	1.00	0.89	1.13	0.966
Health condition	Extremely good, good								
	Normal	1.66	1.51	1.82	<0.0001	1.45	1.26	1.66	<0.0001
	Extremely poor, poor	3.27	2.95	3.64	<0.0001	2.02	1.68	2.42	<0.0001
History of mental illness	No								
	Yes	1.65	1.44	1.89	<0.0001	1.11	0.87	1.41	0.388
ADL (eating, getting dressed and undressed, using the toilet, going shopping, etc.)	Independent								
	Assistance required	6.38	5.37	7.60	<0.0001	3.85	2.84	5.23	<0.0001
K6 score (Psychological distress)	<13								
	≥13	1.95	1.78	2.13	<0.0001	1.16	0.98	1.38	0.084
PCL-S (Trauma reaction scale)	<44								
	≥44	1.44	1.34	1.54	<0.0001	0.98	0.86	1.12	0.780
Frequency of exercise	Almost every day								
	2–4 times a week	0.96	0.89	1.03	0.26	0.91	0.81	1.03	0.124
	About once a week	1.13	1.03	1.24	0.01	1.01	0.87	1.17	0.869
	Rarely or never	3.32	3.05	3.61	<0.0001	2.74	2.40	3.13	<0.0001
AIS score	For every 1-point increase	1.15	1.14	1.17	<0.0001	1.08	1.05	1.10	<0.0001

**Table 3:** Logistic regression analysis of the dependent variables for social nonparticipation among evacuees who relocated to areas within the Fukushima prefecture.

Factor		Crude Odds Ratio	95% Confidence Interval		*p* Value	Adjusted Odds Ratio	95% Confidence Interval		*p* Value
Age	For every 1-year increase	1.03	1.03	1.04	<0.0001	1.02	1.01	1.03	<0.0001
Sex	Men								
	Women	1.20	1.13	1.27	<0.0001	1.07	0.97	1.18	0.1855
Tsunami	Did not experience								
	Experienced	0.89	0.83	0.96	0.002	0.74	0.65	0.84	<0.0001
Result of the domicile earthquake damage assessment	No or some damage								
	Partial destruction	1.06	0.95	1.19	0.316	1.03	0.85	1.24	0.783
	Large-scale partial or complete destruction	1.44	1.28	1.62	<0.0001	1.76	1.44	2.14	<0.0001
Earthquake-related bereavement	No								
	Yes	1.09	1.02	1.18	0.019	1.03	0.91	1.17	0.645
Health condition	Extremely good, good								
	Normal	1.70	1.54	1.88	<0.0001	1.49	1.29	1.73	<0.0001
	Extremely poor, poor	3.40	3.04	3.81	<0.0001	2.02	1.67	2.45	<0.0001
History of mental illness	No								
	Yes	1.73	1.49	2.01	<0.0001	1.23	0.95	1.59	0.120
ADL (eating, getting dressed and undressed, using the toilet, going shopping, etc.)	Independent								
	Assistance required	7.06	5.82	8.57	<0.0001	4.50	3.22	6.28	<0.0001
K6 score (Psychological distress)	<13								
	≥13	2.01	1.82	2.21	<0.0001	1.24	1.03	1.48	0.022
PCL-S (Trauma reaction scale)	<44								
	≥44	1.45	1.35	1.56	<0.0001	0.98	0.85	1.13	0.784
Frequency of exercise	Almost every day								
	Two to four times a week	0.94	0.87	1.02	0.156	0.91	0.80	1.03	0.151
	About once a week	1.17	1.06	1.29	0.003	1.02	0.87	1.19	0.833
	Rarely or never	3.34	3.05	3.67	<0.0001	2.79	2.43	3.21	<0.0001
AIS score	For every one-point increase	1.16	1.14	1.18	<0.0001	1.08	1.05	1.11	<0.0001

**Table 4:** Logistic regression analysis of the dependent variables for social nonparticipation among evacuees who relocated to areas outside the Fukushima prefecture.

Factor		Crude Odds Ratio	95% Confidence Interval		*p* Value	Adjusted Odds Ratio	95% Confidence Interval		*p* Value
Age	For every 1-year increase	1.03	1.02	1.05	<0.0001	1.02	0.99	1.04	0.136
Sex	Men								
	Women	1.38	1.17	1.63	0.000	1.15	0.88	1.51	0.306
Highest education level completed	Elementary school or junior high school								
	High school	0.80	0.65	0.97	0.021	0.80	0.58	1.10	0.167
	Vocational school or junior college	0.87	0.65	1.17	0.354	0.73	0.47	1.13	0.155
	University or graduate school	0.73	0.52	1.03	0.072	0.94	0.57	1.56	0.816
Changes in work	No changes								
	Had changes	0.75	0.63	0.90	0.002	0.80	0.61	1.04	0.093
Health condition	Extremely good, good								
	Normal	1.22	0.92	1.62	0.168	1.12	0.73	1.72	0.595
	Extremely poor, poor	1.99	1.45	2.73	<0.0001	1.57	0.94	2.63	0.088
History of mental illness	No								
	Yes	1.05	0.75	1.49	0.774				
ADL (eating, getting dressed and undressed, using the toilet, going shopping, etc.)	Independent								
	Assistance required	6.38	5.37	7.60	<0.0001	1.97	1.00	3.89	0.050
K6 score (Psychological distress)	<13								
	≥13	1.37	1.09	1.73	0.008	0.92	0.63	1.35	0.673
Frequency of exercise	Almost every day								
	Two to four times a week	1.12	0.91	1.39	0.284	1.09	0.78	1.52	0.607
	About once a week	1.07	0.82	1.40	0.622	1.29	0.85	1.96	0.229
	Rarely or never	3.34	2.60	4.30	<0.0001	2.73	1.88	3.98	<0.0001
AIS score	For every one-point increase	1.07	1.03	1.12	0.002	1.04	0.98	1.11	0.180

**Table 5:** Logistic regression analysis of the dependent variables for social nonparticipation in the activities of daily living (ADL) independent group.

Factor		Crude Odds Ratio	95% Confidence Interval		*p* Value	Adjusted Odds Ratio	95% Confidence Interval		*p* Value
Age	For every 1-year increase	1.02	1.01	1.02	<0.0001	1.02	1.02	1.03	<0.0001
Sex	Men								
	Women	1.18	1.11	1.25	<0.0001	1.12	1.02	1.24	0.017
Highest education level completed	Elementary school or junior high school								
	High school	1.05	0.98	1.12	0.160	0.97	0.88	1.08	0.615
	Vocational school or junior college	1.11	0.99	1.24	0.077	0.93	0.78	1.11	0.434
	University or graduate school	1.31	1.13	1.51	0.000	1.34	1.08	1.65	0.008
Evacuation location	Fukushima prefecture								
	Other prefecture	2.29	2.09	2.51	<0.0001	2.13	1.84	2.47	<0.0001
Tsunami	Did not experience								
	Experienced	0.88	0.82	0.94	0.0002	0.78	0.69	0.87	<0.0001
Result of the domicile earthquake damage assessment	No or some damage								
	Partial destruction	1.10	0.98	1.23	0.097	1.10	0.92	1.31	0.312
	Large-scale partial or complete destruction	1.45	1.30	1.63	<0.0001	1.79	1.49	2.16	<0.0001
Health condition	Extremely good, good								
	Normal	1.62	1.48	1.79	<0.0001	1.46	1.27	1.68	<0.0001
	Extremely poor, poor	2.90	2.59	3.23	<0.0001	2.01	1.67	2.42	<0.0001
History of mental illness	No								
	Yes	1.56	1.34	1.81	<0.0001	1.17	0.91	1.50	0.216
K6 score (Psychological distress)	<13								
	≥13	1.84	1.67	2.02	<0.0001	1.13	0.95	1.35	0.161
PCL-S (Trauma reaction scale)	<44								
	≥44	1.36	1.27	1.46	<0.0001	0.98	0.86	1.12	0.769
Frequency of exercise	Almost every day								
	Two to four times a week	0.95	0.88	1.03	0.245	0.90	0.80	1.01	0.081
	About once a week	1.13	1.03	1.25	0.011	1.02	0.88	1.18	0.813
	Rarely or never	3.14	2.88	3.43	<0.0001	2.74	2.40	3.13	<0.0001
AIS score	For every one-point increase	1.14	1.12	1.16	<0.0001	1.08	1.05	1.10	<0.0001

**Table 6:** Logistic regression analysis of the dependent variables for social nonparticipation in the activities of daily living (ADL) assistance required group.

Factor		Crude Odds Ratio	95% Confidence Interval		*p* Value	Adjusted Odds Ratio	95% Confidence Interval		*p* Value
Age	For every 1-year increase	1.04	1.01	1.07	0.005	1.04	1.01	1.07	0.003
Sex	Men								
	Women	1.26	0.89	1.80	0.198	1.19	0.81	1.76	0.380
Health condition	Extremely good, good								
	Normal	2.19	1.07	4.49	0.033	2.04	0.93	4.47	0.074
	Extremely poor, poor	3.14	1.54	6.42	0.002	3.31	1.51	7.26	0.003
Frequency of exercise	Almost every day								
	Two to four times a week	0.79	0.49	1.29	0.349	0.80	0.48	1.34	0.400
	About once a week	1.04	0.57	1.91	0.906	0.96	0.51	1.79	0.887
	Rarely or never	3.11	1.83	5.29	<0.0001	3.14	1.82	5.42	<0.0001

## Data Availability

The datasets analyzed during the present study are not publicly available because the data of the Fukushima Health Management Survey belongs to the government of Fukushima prefecture and can only be used within that organization.
